# Editorial: Transcriptional and posttranscriptional homeostasis in inflammation and inflammatory diseases

**DOI:** 10.3389/fimmu.2024.1391199

**Published:** 2024-03-06

**Authors:** Xinyi Wang, Yaoxin Liu, Yuanxi Mo, Ning Tan, Wei Huang, Yuliang Feng, Lei Jiang

**Affiliations:** ^1^ Department of Cardiology, Guangdong Cardiovascular Institute, Guangdong Provincial People’s Hospital (Guangdong Academy of Medical Sciences), Guangzhou, China; ^2^ Department of Cardiology, Guangdong Provincial People’s Hospital (Guangdong Academy of Medical Sciences), Southern Medical University, Guangzhou, China; ^3^ Heart, Lung and Vascular Institute, Department of Internal Medicine, Division of Cardiovascular Health and Disease, University of Cincinnati, Cincinnati, OH, United States; ^4^ Department of Pharmacology, School of Medicine, Southern University of Science and Technology, Guangdong, China

**Keywords:** chromatin dynamics, 3D genomics, alternative splicing, inflammation, inflammatory diseases

Inflammation is vital to protect the host against foreign organism invasion and cellular damage. However, unresolved chronic inflammation is a recognized precursor and accelerator of numerous human diseases (1). Immune cells play a wide range of fundamental physiological roles during inflammation and inflammatory diseases, necessitating precise regulation of gene expression (2). Cutting-edge genomic sequencing technologies have advanced the investigation of transcriptional and posttranscriptional processes. Delving into their regulatory mechanisms can enhance our understanding of inflammatory diseases, potentially improving their development, progression and prognosis. This Research Topic encompasses a range of studies investigating transcriptional and post-transcriptional regulation, including DNA and RNA methylation and modification, histone modifications, non-coding RNAs, and 3D chromatin structures. These studies aim to delineate the impact of such regulations on inflammation and its associated diseases.

For instance, Michaël F Michieletto and colleagues. demonstrated that transcription factors GATA-3 and RORα upregulate the Innate lymphoid cells (ILCs)-lineage-defining factor Id2, promoting the specific interactions between Id2 promoter and distal enhancer (3). In addition, Katia Georgopoulo et al. found that IKAROS-bound enhancers could override CTCF-imposed boundaries to assemble lineage-specific regulatory units, which is vital to assemble the correct genome structure needed for B cells differentiation and life-saving antibodies production (4). As for the posttranscriptional regulation, Ledong Wan et al. showed that SRSF1 promoted IL1R1 expression through alternative splicing in the 5’UTR that enhances mRNA stability, finally promoting the development of pancreatitis and pancreatic cancer (5).

Sequencing and gene editing technology development allows researchers to explore based risk genetic variants for common and complex diseases. Identifying the disease-associated regulatory SNP can help researchers find new pathogenic genes and understand how different allele effects histone modification and genes expression (6). In fact, the majority of the identified risk variants are located in non-coding regions of the genome, which makes it difficult to perturb its functionality. Recent studies highlight the role of non-coding regions, which harbor disease-associated SNPs that regulate the transcription of long non-coding RNAs (lncRNAs). For example, Ezio T Fok et al. demonstrated that long noncoding RNA AMANZI activate the transcription of *IL37* through the formation of a dynamic long-range chromatin contact that leads to the temporal delay of anti-inflammatory responses. The common variant rs16944 present in AMANZI augments this regulatory circuit, predisposing individuals to enhanced proinflammation or immunosuppression (7). Therefore, exploring the interaction between risk SNP and diseases can promote the development of precision medicine and personalized medical healthcare (8).

The field of epigenetics has rapidly evolved over the last decade, several epigenetic drugs have been introduced into the clinic to treat cancer, and many more are being investigated in clinical trials. Epigenetic drugs that have been approved by the FDA includes DNA methylation agents, chromatin remodelers specially HDACs, and noncoding RNAs (9–11). Given the relationship between inflammation and cancer, it is necessary to explore the role of these drugs in anti-inflammation. HMGB1 plays the complex roles in the development of many diseases such as autoimmune diseases and cancers. Konstantinos Sofiadis et al. recently showed that a considerable number of Topologically Associating Domain (TAD) boundaries in proliferating human cells are marked by HMGB2 and these boundaries are remodeled upon the nuclear loss of HMGB2. HMGB1 lost could deregulate the inflammatory activation-related genes located in these loop domains (12). Naoyuki Senda et al. examined the H3K4me3 marks over the whole genome in the PAM212 (mouse keratinocyte cell line), and found that *Hmgb1*-deficient keratinocytes showed increased H3K4me3 marks near the transcription start site of the *Il24* gene (R1) and the other at the distal site (R2). These changes in H3K4me3 marks increased *Il24* mRNA expression and promotes skin inflammation, which suggested that HMGB1-mediated chromatin remodeling can attenuate *Il24* gene expression to protect allergic contact dermatitis (13–15).

In summary, the published manuscripts in this Research Topic have provided an interesting overview of the roles of transcriptional and posttranscriptional regulation in inflammation and inflammatory diseases. The exploration of these regulatory aspects may lead to novel treatments, advancing precision medicine and personalized healthcare. Further research in this domain is essential, and it is hoped that these findings will inspire and inform future scientific endeavors.

## Author contributions

XW: Writing – original draft. YL: Resources, Writing – original draft. YM: Software, Validation, Writing – original draft. NT: Supervision, Writing – original draft. WH: Writing – review & editing. YF: Writing – review & editing. LJ: Writing – review & editing.
